# Extracellular Matrix Tissue Patch for Pulmonary Artery Repair in Pediatric Cardiac Surgery: A Single-Center Experience

**DOI:** 10.3390/jcm15031177

**Published:** 2026-02-03

**Authors:** Marcin Gładki, Paweł R. Bednarek, Anita Węclewska, Tomasz Urbanowicz, Anna Olasińska-Wiśniewska, Marek Jemielity

**Affiliations:** 1 Department of Pediatric Cardiac Surgery, Poznan University of Medical Sciences, 60-572 Poznan, Poland; 2 Department of Cardiac Surgery and Transplantology, Poznan University of Medical Sciences, 61-848 Poznan, Poland

**Keywords:** pediatric cardiac surgery, congenital heart surgery, congenital heart defect, pulmonary artery repair, extracellular matrix patch

## Abstract

**Introduction**: Congenital structural anomalies of the pulmonary artery in children, encompassing defects such as pulmonary atresia (PA), pulmonary stenosis (PS), pulmonary artery hypoplasia, and tetralogy of Fallot (ToF), pose significant challenges in pediatric cardiac surgery due to impaired blood flow in pulmonary circulation. Traditional options for conventional repair—including autologous materials such as the native pericardium and synthetic materials such as artificial patches—have limitations including a lack of growth potential and vulnerability to restenosis over time. ProxiCor^®^ patches, based on the extracellular matrix (ECM), have emerged as biologically compatible substitutes capable of fostering tissue regeneration. The primary outcomes of this study were the safety (absence of patch-related complications such as restenosis, dilation, aneurysm, infection, or thrombosis) and feasibility (intraoperative handling and surgical success) of ProxiCor^®^ for pulmonary artery and right ventricular outflow tract (RVOT) reconstruction in a single-center pediatric cohort. Secondary outcomes included mortality, postoperative complications (prolonged mechanical ventilation > 72 h, need for continuous renal replacement therapy (CRRT), and intensive care unit (ICU) and hospital stay), and qualitative echocardiographic assessment of vessel patency during follow-up. **Patients and methods**: A retrospective analysis was conducted in 25 consecutive pediatric patients who underwent pulmonary artery or RVOT reconstruction with ProxiCor^®^ at the Department of Pediatric Cardiac Surgery in Poznań (Poland) between the years 2023 and 2024. Surgical techniques, clinical outcomes, and follow-up data were assessed using transthoracic echocardiography (TTE). **Results**: The median age was 224 (Q1–Q3: 124–362) days, and median weight was 4.2 (Q1–Q3: 2.8–8.5) kg. Procedures targeted repairs of the main pulmonary artery (MPA), right pulmonary artery (RPA), left pulmonary artery (LPA), and RVOT. Diagnoses included tetralogy of Fallot (ToF), pulmonary artery stenosis (PS), pulmonary atresia (PA), pulmonary artery hypoplasia, and anomalous left coronary artery from the pulmonary artery (ALCAPA). The mortality rate stood at 8% (2/25), stemming from multiorgan failure and hemorrhagic stroke, unrelated to the patch. Over a median observation period of 483 (Q1–Q3: 363–584) days, no patch-related complications (e.g., restenosis or dilation) arose. The median hospitalization time was 22 (Q1–Q3: 8.5–38.5) days. **Conclusions**: ProxiCor^®^ ECM patches appear to be safe and feasible for use in pulmonary artery and RVOT reconstruction, with favorable early outcomes. However, the small cohort size, lack of a control group, and limited mid- to long-term echocardiographic data preclude definitive conclusions about long-term outcomes or comparative effectiveness.

## 1. Introduction

Congenital defects of the pulmonary artery include a variety of structural abnormalities that compromise the pulmonary vasculature system, impairing blood flow from the right ventricle to the pulmonary circulation. These conditions include (1) pulmonary atresia (PA), (2) pulmonary stenosis (PS), (3) pulmonary artery hypoplasia, and (4) tetralogy of Fallot (ToF). PA is characterized by the complete absence or closure of the pulmonary valve (due to embryological non- or underdevelopment), limiting direct communication between the right ventricle and pulmonary arteries. PS involves narrowing of the pulmonary valve or artery, obstructing blood flow and increasing pressure in the right ventricle. Pulmonary artery hypoplasia refers to the underdevelopment of the pulmonary artery whereby direct communication between the right ventricle and pulmonary arteries is severed, which restricts lung perfusion and further oxygenation. PS involves narrowing of the pulmonary valve or artery, obstructing blood flow and increasing chamber pressure in the right ventricle. ToF, a complex defect, combines PS with a ventricular septal defect (VSD), right ventricular hypertrophy, and an overriding aorta (positioned over both ventricles), leading to cyanosis due to mixing of oxygenated and deoxygenated blood. These defects vary in symptomatic severity and clinical presentation, frequently requiring advanced imaging diagnostics and surgical interventions to restore adequate pulmonary circulation flow and enhance patients’ outcomes.

Congenital defects of the pulmonary artery often require patch augmentation to restore adequate blood flow. Traditional materials have significant limitations that drive the need for improved alternatives. The autologous pericardium, while biocompatible and readily available, frequently undergoes fibrosis, shrinkage, or aneurysmal degeneration over time. Synthetic patches (e.g., ePTFE/Gore-Tex^®^ or Dacron^®^) lack growth potential and are prone to infection, calcification, and restenosis in growing children, often necessitating reoperations. Homografts offer durability but are limited by availability, immunogenicity, and progressive degeneration.

Porcine small intestinal submucosa-derived extracellular matrix (SIS-ECM) patches, such as CorMatrix^®^ (the predecessor of the material considered in this study), showed initial promise due to potential for host cell infiltration and tissue remodeling. However, clinical studies reported mixed outcomes, including inflammatory responses, fibrosis, and higher reintervention rates in certain applications (e.g., valve reconstruction or high-pressure sites). ProxiCor^®^ (Elutia, Inc., Gaithersburg, MA, USA) is a non-crosslinked, multi-layer SIS-ECM scaffold designed to improve biocompatibility and regenerative potential compared to earlier crosslinked versions; however, evidence for its use in pediatric pulmonary artery reconstruction remains limited, prompting this preliminary assessment.

Different patch materials used in congenital heart surgery have been evaluated, and both their benefits and drawbacks have been highlighted [1]. Nevertheless, evidence regarding the clinical use of ProxiCor^®^—a porcine small intestinal submucosa (SIS)-derived extracellular matrix product—in pediatric patients with structural abnormalities of the pulmonary artery remains scarce.

This goal of this single-center retrospective analysis was to assess the applicability and early outcomes of decellularized extracellular matrix scaffolds for pulmonary artery reconstruction in children using data from the national cardiac surgery registry (pol. Krajowy Rejestr Operacji Kardiochirurgicznych—KROK, Children’s Memorial Health Institute, Warsaw, Poland; https://krok.csioz.gov.pl/krok/, accessed on 11 January 2025).

## 2. Patients and Methods

### 2.1. Patients

This study included 25 consecutive pediatric patients who underwent surgical procedures on the pulmonary artery and/or its branches or right ventricular outflow tract (RVOT) at the Department of Pediatric Cardiac Surgery, Poznan University of Medical Sciences (Poznań, Poland) between 2023 and 2024, following the introduction of ProxiCor^®^ for Cardiac Tissue Repair (CTR) (Elutia, Inc., Gaithersburg, MA, USA; formerly Aziyo Biologics, Inc., Roswell, NM, USA) into the portfolio of available implantable patches. Patients were selected sequentially based on the need for pulmonary artery or RVOT reconstruction using ProxiCor^®^. Exclusion criteria included patients requiring pulmonary valve replacement with a valved graft, those with incomplete medical records, or those undergoing procedures not involving ProxiCor^®^ patches.

Patients were stratified into three mutually exclusive subgroups based on the hemodynamic nature of the primary diagnosis of congenital heart defect (CHD) and the scope and extent of the surgical procedure: (1) pulmonary artery group, (2) RVOT group, and (3) combined group.

The pulmonary artery group included patients with pulmonary artery hypoplasia, PA, PS, and ALCAPA. In this group, ProxiCor^®^ patches were used to enlarge narrowed segments of the pulmonary trunk or its branches, thereby improving vessel diameter and flow continuity. Regarding hypoplastic branches, patches bridged underdeveloped areas while, in cases of stenosis, they were applied to augment narrowed zones and relieve obstruction.

The RVOT group included patients with tetralogy of Fallot (ToF) requiring RVOT enlargement. In this group, patches were used exclusively for RVOT widening to relieve subvalvular, valvular, and/or supravalvular obstruction without the need for valve replacement.

The combined group included patients with ToF who required reconstruction of both the pulmonary arteries and the RVOT due to complex defects involving both structures. In this group, surgical repair addressed multifaceted anomalies, reinforcing both the outflow tract and arterial structures in high-flow settings, such as after shunt revisions or during complete ToF corrections.

This classification reflects the varying hemodynamic stresses encountered: low-pressure pulmonary reconstructions in the first group, ventricular-level repairs in the second, and integrated approaches in the third, thereby enabling precise surgical strategies and outcome evaluation.

### 2.2. Methods

Baseline characteristics, periprocedural clinical and imaging data, and complications were recorded in a dedicated database. Pre- and postprocedural transthoracic echocardiographic (TTE) data, along with baseline, immediate postoperative, and follow-up survival, were also compared. During surgery, transesophageal echocardiography (TEE) was performed. The overall surgery time, cardiopulmonary bypass (CPB) time, aortic cross-clamp (AoX) time, and mechanical ventilation time were analyzed. Prolonged mechanical ventilation was defined as the use of mechanical ventilation for over 72 h. The requirement for continuous renal replacement therapy (CRRT) was recorded. Patients underwent a short-term follow-up between 3 and 6 months post-surgery.

All surgeries were performed from a standard access via median sternotomy using extracorporeal circulation (ECC). The arterial cannula was placed in the aorta. Venous cannulation was performed using two cannulas as follows: the first, the straight cannula, was inserted through the right atrium (RA) into the superior vena cava (SVC); and the second, the curved Pacifico cannula, was placed directly into the inferior vena cava (IVC). If needed, the heart was stopped with cold blood del Nido cardioplegic solution. Whenever the anatomy of the defect permitted, the surgical procedure was performed on the beating heart without cardioplegic arrest.

Reconstruction of the pulmonary arteries (both the trunk and its branches, stemming from anatomical variances) to fill in the missing tissue and expand the diameter of the pulmonary arteries was carried out with a ProxiCor^®^ patch, which was properly prepared for use beforehand.

The ProxiCor^®^ scaffold is delivered as a pre-formed rectangular patch composed of four extracellular matrix (ECM) layers. The patch in dry state possesses inherent sufficient stiffness, enabling accurate shaping using surgical scissors without fraying on tissue edges. After the preparation phase and prior to implantation, the patch requires immersion in saline (or, alternatively, in extravasated blood directly in the surgical field).

Surgeons occasionally thinned the material by peeling off one or more layers when dealing with markedly fragile or underdeveloped vessels in neonates presenting with the most severe end of the spectrum of pulmonary artery hypoplasia and pulmonary atresia (PA). This modification produces a more pliable patch tailored to the specific mechanical anatomical demands of the individual. The decision to delaminate was made intraoperatively, based on the surgeon’s assessment, with the goal of optimizing conformity and integration in anatomically restricted or low-pressure hemodynamic settings.

Before implantation, the patch was consistently soaked in isotonic 0.9% sodium chloride (NaCl) saline solution to regain flexibility, which simplified intraoperative handling and promoted smooth adaptation to adjacent tissues without the risk of sharp-angled folds. This hydration step was applied consistently across all patches, irrespective of any further modifications.

In patients undergoing RVOT reconstruction, the ProxiCor^®^ patch was used in cases not requiring pulmonary valvuloplasty or requiring only pulmonary valve commissurotomy. Patients with significant impairment of pulmonary valve geometry and function required correction with a valved graft, and were thus not included in this study. When performing RVOT reconstruction, the principle of the smallest possible scope of ventriculotomy was respected to minimize the risk of postoperative right ventricular failure. The RVOT incision was guided vertically, starting just below the pulmonary artery valve. Then, after excision of a portion of the hypertrophied musculature obstructing ventricular outflow, the area was widened with a ProxiCor^®^ tissue patch.

The preferred surgical method for sewing the patch to native structures was a continuous running (commonly referred to as the ‘baseball’) technique using a 5-0 USP (size defined by the United States Pharmacopeia (USP) standards) monofilament suture. In neonates with diminutive pulmonary arteries (for both left and/or right pulmonary artery reconstruction), 6-0 USP monofilament sutures were used.

In select cases with intricate vascular anatomy, such as severe hypoplasia or post-banding distortions, additional techniques such as end-to-end anastomosis augmentation or longitudinal arteriotomy were integrated with patch placement to optimize flow dynamics and prevent turbulence. Intraoperative echocardiography was employed to guide adjustments to ensure immediate hemodynamic stability post-repair.

### 2.3. Statistical Analysis

The data distribution was checked using the Shapiro–Wilk test. As the variables deviated from normality, the results are given as medians with interquartile ranges (Q1–Q3). Categorical parameters are reported as absolute counts and percentages. Analyses were conducted with the Jeffreys’s Amazing Statistics Program (JASP) statistical software (version 0.13.1, JASP Team, Amsterdam, The Netherlands, 2020).

The consent of individual patients was waived due to the retrospective nature of the study, as determined by the institutional bioethics committee board.

### 2.4. Outcomes and Follow-Up

Primary outcomes: Safety (patch-related complications: restenosis, dilation/aneurysm, thrombosis, and infection) and feasibility (intraoperative handling, suture retention, and conformity).

Secondary outcomes: Mortality (early ≤30 days, late >30 days), postoperative morbidity (prolonged ventilation > 72 h, CRRT need, ICU/hospital stay), and qualitative transthoracic echocardiography (TTE) assessment of repaired segments (patency, absence of obstruction or dilation). Follow-up TTE was performed routinely at discharge, with further postoperative observation scheduled according to individual indications, with longer intervals as clinically indicated.

No concurrent or historical control group was included, as this was a retrospective feasibility study of a newly introduced material (ProxiCor^®^, available in our center since 2023). Patients were selected consecutively based on clinical need for patch reconstruction (excluding valved conduits).

## 3. Results

The study population consisted of 25 patients, including 17 males (68%) and 8 females (32%), with a median age of 224 (Q1–Q3: 124–362) days; comprising 4 newborns (≤28 days) with a median age of 18 (Q1–Q3: 15.8–21.3) days, 15 infants (1–12 months) with a median age of 217 (Q1–Q3: 171.5–270.5) days, and 6 children (>12 months) with a median age of 2442 (Q1–Q3: 888–3091) days. The median height and weight were 68 (Q1–Q3: 62–74) cm and 6.7 (Q1–Q3: 5.1–8.5) kg, respectively.

**Figure 1:**
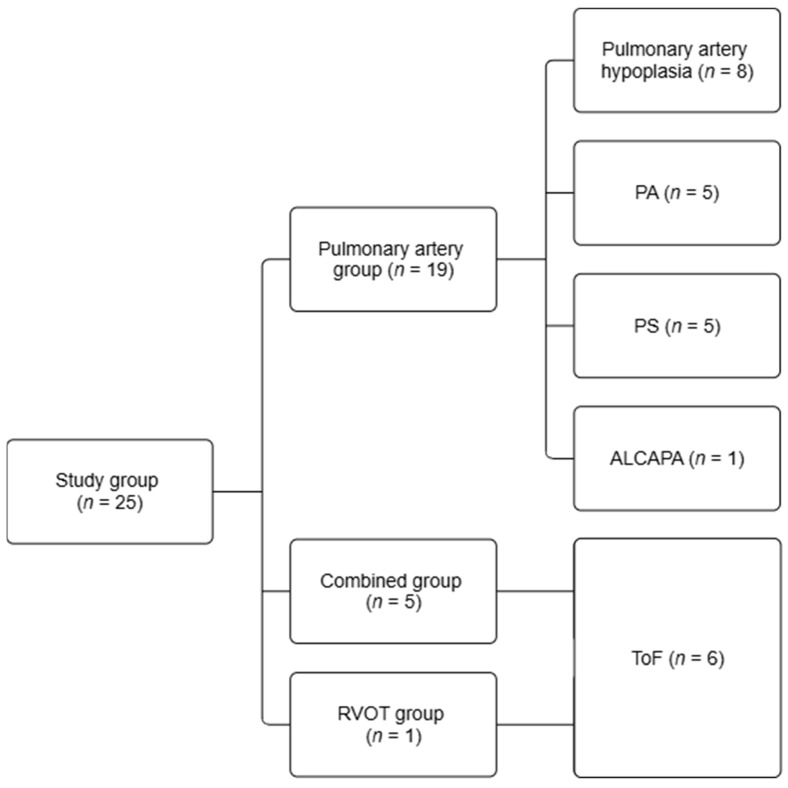
Patient subgroup classification. Abbreviations: ALCAPA—anomalous left coronary artery from the pulmonary artery (Bland–White–Garland syndrome), PA—pulmonary atresia, PS—pulmonary stenosis, RVOT—right ventricular outflow tract, ToF—tetralogy of Fallot.

Of the 25 patients, 19 underwent procedures involving the pulmonary trunk/main pulmonary artery (MPA) and/or its branches as follows: in cases involving the right pulmonary artery (RPA) and/or left pulmonary artery (LPA), 1 patient underwent isolated RVOT reconstruction, while 5 patients underwent combined pulmonary artery and RVOT procedures. The pulmonary artery group (*n* = 19) included patients with pulmonary artery hypoplasia (*n* = 8, 42%), PA (*n* = 5, 26%), PS (*n* = 5, 26%), and ALCAPA (*n* = 1, 5%). The RVOT group (*n* = 1, 100%) included 1 patient with ToF. The combined group (*n* = 5, 100%) consisted of patients with ToF, all of whom required both pulmonary artery and RVOT reconstruction due to complex defects involving both structures. The details of the patient subgroups are presented in Figure 1.

No significant differences in early postoperative outcomes (e.g., mortality, ICU stay, or ventilation time) were observed across subgroups, although the small sample size and heterogeneity of defects limited definitive subgroup comparisons.

10 patients underwent elective reoperations as part of multi-staged surgical treatment, with prior procedures including 5 systemic-to-pulmonary shunt procedures; 3 Norwood procedures for hypoplastic left heart syndrome (HLHS); 3 anatomical corrections of transposition of the great arteries (TGA), including 2 Jatene arterial switch operations and 1 Nikaidoh aortic root translocation for coexisting left ventricular outflow tract obstruction repair; 2 Muller–Dammann pulmonary artery banding (PAB) procedures; and 1 RVOT reconstruction. The sum of prior procedures (14) exceeded the number of reoperated patients (10) as some patients underwent multiple reoperations.

The demographic and clinical characteristics of the patients are detailed in Table 1.

All patients underwent routine postoperative transthoracic echocardiography (TTE) with a median follow-up of 483 (Q1–Q3: 363–584) days (from the first patch application in the first patient in 2023 up to the completion of the statistical analysis in 2025). Patients were enrolled sequentially, leading to varying follow-up lengths based on operation timing, with earlier cases monitored for nearly two years and more recent cases for shorter periods. TTE assessments focused primarily on the immediate postoperative period, confirming no evidence of patch-related complications such as restenosis or dilation in the repaired pulmonary artery or RVOT segments, although these findings are preliminary due to the small cohort and lack of a control group.

All 25 (100%) procedures were performed using a median sternotomy with a cardiopulmonary bypass (CPB). The median CPB and aortic cross-clamp (AoX) times were 100 (Q1–Q3: 73–142) minutes and 68 (Q1–Q3: 59–72) minutes, respectively. In 9 (36%) patients, the heart function was routinely stopped using a protocol with cold blood del Nido cardioplegic solution.

The median postoperative stay in the intensive care unit (ICU) was 251 (Q1–Q3: 106–825) hours. The median mechanical ventilation time was 173 (Q1–Q3: 26–484) hours, with 16 (64%) patients requiring prolonged mechanical ventilation support (over 72 h). Postoperative acute kidney failure requiring continuous renal replacement therapy (CRRT) using continuous veno-venous hemodiafiltration (CVVHDF) protocol was noted in 2 (8%) cases, as presented in Table 2.

The early and overall mortality in the presented group was 2 (8%) patients. The causes of death were postoperative multiorgan failure in 1 (4%) patient and hemorrhagic stroke in 1 (4%) patient, neither of which was directly linked to the ProxiCor^®^ patch performance. The 8% mortality rate aligns with reported rates for complex congenital heart defect (CHD) repairs, though direct comparisons are limited by variations in patient complexity and surgical techniques. Both deaths occurred in neonates with complex pre-existing conditions (1 with HLHS post-Norwood procedure and 1 with ToF and prior systemic-to-pulmonary shunt). While no direct patch-related complications were observed, the retrospective design and lack of detailed perioperative data (e.g., inflammatory markers or hemodynamic stability) limit the ability to rule out indirect effects of the patch—such as subtle inflammatory responses or altered hemodynamics—on these outcomes, particularly in high-risk neonates. The median hospitalization time was 22 (Q1–Q3: 8.5–38.5) days, as summarized in Table 3.

TTE parameters: Due to the retrospective design and heterogeneous anatomy (many with prior shunts/interventions), the nature of the echocardiographic data precluded uniform consistent quantitative analysis; thus, qualitative assessment was performed. Routine postoperative TTE (immediate and follow-up) confirmed qualitative patency without evidence of significant obstruction, restenosis, or dilation in any patient. Where measured, no elevated gradients suggestive of patch failure were noted.

## 4. Discussion

The use of ProxiCor^®^ ECM patches for pulmonary artery and RVOT reconstruction in pediatric cardiac surgery represents a promising advancement in addressing CHD such as PA, PS, pulmonary artery hypoplasia, and ToF. This single-center retrospective study provides preliminary evidence in a small cohort of 25 pediatric patients suggesting that ProxiCor^®^ patches may be safe and feasible; although the retrospective design, small sample size, and lack of a control group limit the generalizability of these findings. These findings align with the growing interest in biological scaffolds that offer biocompatibility and potential for tissue remodeling, addressing key limitations of traditional materials such as the autologous pericardium and synthetic patches. In comparison to established methods, earlier approaches to pulmonary artery anomalies predominantly utilized the autologous pericardium or synthetic polymers like those based on expanded polytetrafluoroethylene (ePTFE) such as Gore-Tex^®^ (W.L. Gore & Associates, Inc., Newark, DE, USA) or polyethylene terephthalate (PET) such as Dacron^®^ (DuPont Corporation, Wilmington, DE, USA) [1,2]. However, these materials have been increasingly sidelined due to their inflexibility, propensity for abrupt bending causing flow disruptions, and absence of expansion capability, leading to frequent narrowing or calcification in growing children and potentially necessitating replacement. The autologous pericardium, while initially pliable, often undergoes fibrosis or aneurysmal changes under prolonged exposure to pulmonary pressures [1]. Additionally, pulmonary homografts, which are commonly used as patch materials in pulmonary artery reconstruction, offer good durability but are limited by availability [3] and their potential for immune-mediated degeneration, calcification, and lack of growth, similar to synthetic materials [2,4,5]. In our experience, ProxiCor^®^ exhibited no such issues over the follow-up, although extended monitoring is vital to evaluate sustained performance, and direct comparisons with homografts in larger cohorts are warranted. ProxiCor^®^ showed no evidence of restenosis or dilation in early postoperative TTE assessments in this cohort; however, the absence of a control group (e.g., synthetic patches, the autologous pericardium, or pulmonary homografts) and limited mid- to long-term data prevent direct comparisons or conclusions about sustained performance.

Compared to pulmonary homografts, which provide durable vascular tissue with low thrombogenicity but are limited by donor availability, potential immunogenicity, and a lack of growth potential in pediatric patients [3,6,7], ECM patches such as ProxiCor^®^ offer advantages in terms of off-the-shelf availability, biocompatibility, and the potential for host tissue remodeling and integration. While homografts have shown favorable mid-term outcomes in pulmonary artery reconstruction [3], their use is often reserved for more complex reconstructions due to procurement challenges, whereas ECM patches may serve as a versatile alternative for patch augmentation in growing children [8]. Recent mid-term data from retrospective cohorts also indicate comparable outcomes across various patch materials in PA augmentation, with reintervention rates influenced more by patient anatomy and prior interventions than by patch type itself [9].

One of the primary advantages of ProxiCor^®^ ECM patches is their potential to integrate with native tissue and support remodeling, which is particularly critical in pediatric patients whose cardiovascular structures are still growing. Unlike synthetic patches—which are prone to restenosis, calcification, and a lack of growth potential—or the autologous pericardium—which may become fibrotic or aneurysmal over time—ECM patches derived from porcine SIS may facilitate cellular infiltration and tissue regeneration. However, as no patches were explanted, the absence of histological data in this study significantly limits our understanding of how ProxiCor^®^ biologically interacts with host tissue. Histological analysis could reveal critical details about cellular infiltration, neovascularization, extracellular matrix remodeling, and potential inflammatory responses, which are essential to confirming the regenerative potential of ECM patches. Previous studies on SIS-ECM patches, such as CorMatrix^®^, have reported variable degrees of tissue integration, with some showing robust cellular repopulation and others noting inflammatory or fibrotic responses [10,11,12]. Without histological data, we cannot confirm whether ProxiCor^®^ supports sustained tissue remodeling or elicits subtle inflammatory responses that could influence long-term outcomes, particularly in neonates with fragile vasculature or complex defects. Future studies incorporating biopsy or explant analysis, where feasible, are critical to elucidating these biological interactions. No instances of restenosis or patch dilation were observed in the immediate postoperative period in this study, as confirmed via intraoperative and routine postoperative TTE assessment during follow-up. This qualitatively demonstrated that vessel patency was maintained and provided evidence for a lack of narrowing or aneurysmal changes in the repaired segments, suggesting that ProxiCor^®^ maintains structural integrity in the short-term. However, the lack of consistent mid- to long-term echocardiographic follow-up limits our ability to evaluate the restenosis rate over time or to compare preoperative and postoperative pressure gradients across the treated pulmonary artery or RVOT segments. Moreover, the variability in follow-up durations (Q1–Q3: 363–584 days) due to sequential enrollment introduces potential bias, as patients with shorter follow-up may not yet exhibit late complications like restenosis or aneurysm formation, which could skew the interpretation of early outcomes. Such data would be critical to assessing the long-term efficacy of ProxiCor^®^ in preventing restenosis and maintaining hemodynamic improvements, particularly in patients with stenotic or hypoplastic pulmonary tracts. The absence of histological data further restricts our understanding of tissue remodeling and cellular infiltration, which is essential for confirming the regenerative potential of ProxiCor^®^. Long-term studies with histological analysis and extended echocardiographic follow-up are needed to verify sustained growth, adaptation, and hemodynamic performance as the child develops.

The potential of ProxiCor^®^ aligns with advancements in biofabrication, particularly in the development of interactive biomaterials for congenital cardiac surgery. As noted in the recent literature [4], biomaterials such as SIS-ECM are designed to act as scaffolds that support recipient cell infiltration and tissue regeneration, addressing the critical need for growth potential in pediatric patients. Unlike inert synthetic materials such as ePTFE, which remain static and are associated with complications including calcification and thrombosis, SIS-ECM patches like ProxiCor^®^ are biodegradable and interactive, promoting remodeling through metabolic rather than inflammatory processes. This characteristic is particularly advantageous in pulmonary artery repairs, where the patch must withstand dynamic hemodynamic stresses while integrating with growing tissues. While the absence of patch-related complications in this small cohort is encouraging, the lack of a control group and the small sample size limit conclusions about whether ProxiCor^®^ could reduce reoperation rates compared to other materials; this remains a significant challenge in CHD management, where approximately 15% of patients require further surgeries due to implant failure or outgrowing repairs.

The surgical techniques employed in this study—including the use of continuous running sutures and occasional delamination of the patch for neonates—highlight the adaptability of ProxiCor^®^ to diverse anatomical challenges. The ability to delaminate the patch for use in fragile neonatal tissues is particularly noteworthy, as it addresses the mechanical demands of hypoplastic pulmonary arteries while maintaining ease of handling. The consistent use of a hydration step with isotonic saline further enhances the patch’s conformability, reducing the risk of kinking or poor integration with native structures. These technical considerations are critical in pediatric cardiac surgery, where precise tailoring to small and delicate vasculature is essential. No substantial differences in clinical outcomes were observed between standard and delaminated patches, although the latter were more frequently used in neonates due to tissue fragility.

Biofabrication strategies, such as those explored in recent studies [4,13], further underscore the potential of ECM-based patches such as ProxiCor^®^. The use of acellular constructs, as seen with ProxiCor^®^, offers advantages in clinical translation due to ease of storage, reduced immunological risks, and off-the-shelf availability, which are critical for urgent interventions in neonates. However, the lack of cellular components in acellular patches like ProxiCor^®^ limits their growth potential compared to cell-laden constructs, which could theoretically incorporate patient-derived stem cells to enhance tissue regeneration and long-term integration. While our study demonstrates the short-term efficacy of acellular ProxiCor^®^ patches, future research could explore the integration of cellular biofabrication techniques (e.g., 3D bioprinting) to create patient-specific patches with enhanced growth potential, potentially revolutionizing outcomes in congenital cardiac surgery.

The integration of biofabrication insights also highlights the potential for ProxiCor^®^ to serve as a platform for future advancements; for instance, the development of biodegradable scaffolds—such as those made from polylactic-polyglycolic acid or polydioxanone, which degrade within 2–3 years—could complement the use of SIS-ECM patches by providing temporary structural support while promoting native tissue growth. Such approaches could further reduce the reoperation burden—a critical consideration, given that 10 patients in our cohort underwent staged surgical management. Additionally, the exploration of patient-specific 3D-printed patches or conduits, as discussed in the biofabrication literature [4], could enhance the precision of pulmonary artery repairs; this would be particularly notable in complex cases such as pulmonary atresia, where anatomical variability is significant.

The 8% mortality rate reported in this study is consistent with expected outcomes for complex CHD repairs, particularly in neonates and infants with significant comorbidities, with both deaths occurring in neonates with complex pre-existing conditions (1 with HLHS post-Norwood procedure and 1 with ToF and a prior systemic-to-pulmonary shunt). While no direct patch-related complications were observed, the retrospective design and limited perioperative data (e.g., inflammatory markers and detailed hemodynamic profiles) preclude definitive conclusions about whether the patch indirectly influenced the observed outcomes, such as through subtle inflammatory responses or altered hemodynamics in these high-risk patients. For instance, neonates with HLHS or ToF often face significant hemodynamic instability, and any biomaterial—even if biocompatible—could theoretically contribute to systemic inflammation or affect flow dynamics in ways not captured via TTE. The high rate of prolonged mechanical ventilation (64%) and CRRT use (8%) in this cohort further highlights the complexity of treating these patients, suggesting that factors such as preoperative comorbidities, surgical complexity, and postoperative management likely played a larger role in outcomes than the patch itself. However, without detailed inflammatory or hemodynamic data, we cannot fully exclude subtle indirect effects of ProxiCor^®^ on morbidity or mortality, particularly in neonates. The median hospitalization time of 22 days and prolonged mechanical ventilation in 64% of patients reflect the complexity of the patient cohort and the extensive nature of the procedures, many of which were reoperations. These findings underscore the need for careful patient selection and perioperative management to optimize outcomes. TTE was effective for assessing postoperative outcomes and detailed insights into patch integration and hemodynamic performance over time, but the lack of consistent mid- to long-term TTE data limits our ability to fully evaluate long-term hemodynamic outcomes such as restenosis rates or changes in pressure gradients across the treated segments.

In patients with ToF, surgical repair involves both RVOT reconstruction and VSD closure, which can pose additional risks for postoperative complications. While the former may impair contractility (particularly in the immediate postoperative period), the latter can be associated with an increased risk of atrioventricular block (AVB). A study conducted at our institution [14] investigated the role of preoperative neutrophil counts as a predictive factor for postoperative AVB in pediatric cardiac surgery. As a result, a neutrophil count below 2.59 K/µL was identified as a significant predictor of AVB, with 100% sensitivity and 65.62% specificity. This is particularly relevant for ToF patients, as VSD repair—which is often performed concurrently with RVOT reconstruction—is associated with a higher risk of AVB due to potential disruption of the cardiac conduction system. While our current study did not specifically assess preoperative neutrophil counts, the absence of reported AVB complications in our cohort may suggest that the use of ProxiCor^®^ patches in RVOT reconstruction did not contribute to conduction disturbances, although this observation is based on a small cohort and requires validation in larger, prospective studies. While the 5 ToF patients in the combined group (i.e., those who underwent both pulmonary artery and RVOT reconstruction) showed no patch-related complications, their complex anatomy and prior interventions (e.g., systemic-to-pulmonary shunts in some cases) may have contributed to prolonged ventilation and ICU stays, highlighting the need for further studies to assess patch performance in this high-risk subgroup. Given the limited sample size and the multifactorial nature of postoperative AVB, as reported in the literature [15,16], further research is needed to explore any potential impact of ECM patches on conduction outcomes. However, the findings highlight the potential utility of preoperative neutrophil counts as a simple, accessible biomarker to identify patients at risk for AVB; particularly in those undergoing complex procedures like ToF repair. Future studies integrating inflammatory markers, such as neutrophil counts, could enhance risk stratification and perioperative management in patients undergoing pulmonary artery and RVOT reconstruction, potentially reducing the incidence of conduction-related complications.

The complexity of postoperative management in pediatric cardiac surgery, particularly for patients with prior Norwood procedures for HLHS (as seen in 3 patients in our cohort), underscores the importance of monitoring acid–base balance (ABB) parameters to maintain hemodynamic stability. Another study [17] analyzed ABB parameters (pH, pCO_2_, pO_2_, HCO_3_^−^, base excess, and lactic acid) in neonates post-Norwood procedure, reporting significant correlations between these parameters and clinical outcomes including the duration of mechanical ventilation and hospitalization time. Elevated lactic acid levels, which are indicative of tissue hypoperfusion, were particularly associated with prolonged recovery periods. In our cohort, the prolonged mechanical ventilation observed in 64% of patients and the median hospitalization time of 22 days suggest similar hemodynamic challenges, particularly in neonates and infants with complex defects such as ToF or prior Norwood procedures. Patients with pulmonary artery hypoplasia (*n* = 8) and PA (*n* = 5) in the pulmonary artery group faced significant preoperative hemodynamic challenges due to restricted pulmonary blood flow, which may have contributed to prolonged ventilation and ICU stays, although no direct patch-related complications were observed. The single ALCAPA patient also required prolonged ventilation, likely due to the complexity of coronary reimplantation alongside pulmonary artery repair. Although ABB parameters were not directly measured in our study, the findings emphasize the need for meticulous postoperative monitoring of ABB to optimize pulmonary and systemic blood flow balance, which is critical in patients undergoing pulmonary artery and RVOT repairs. Integrating ABB monitoring into the perioperative care of such patients could further enhance outcomes by guiding adjustments in ventilation and pharmacotherapy, particularly in high-risk subgroups with a history of staged surgical interventions.

Compared to the existing literature, the results of this study are encouraging but preliminary. Previous studies on ECM patches—such as those using CorMatrix^®^ (another SIS-derived ECM product)—have reported mixed outcomes, with some noting early promise but others highlighting concerns about their long-term durability and inflammatory responses in certain contexts. The findings of this study also raise important considerations for future research. The potential for ECM patches to reduce the need for reoperations—a significant burden in patients with CHD—warrants further investigation. Given that 10 patients in this cohort underwent reoperations as part of staged surgical management, the role of ProxiCor^®^ in reducing subsequent interventions could have significant clinical and economic implications. Future studies should also explore the application of ECM patches in other CHD repairs—such as aortic arch reconstruction or valve repair—in order to assess their broader utility, although such investigations will require larger, controlled studies to establish comparative efficacy. Future studies should also focus on collecting serial echocardiographic data at standardized intervals post-surgery to assess restenosis rates and compare preoperative and postoperative pressure gradients across the stenotic or hypoplastic pulmonary tracts, thus providing a more robust evaluation of long-term patch performance.

Quantitative serial TTE data (e.g., pressure gradients and Z-scores) were not consistently available due to retrospective nature and variable preoperative anatomy. This limits precise hemodynamic assessment, but qualitative imaging showed stable repairs. Prospective protocols with standardized TTE measurements are planned.

### Study Limitations

This retrospective, single-center study is limited by its small cohort size (*n* = 25), which reflects the rarity and heterogeneity of the congenital heart defects treated. The median follow-up is insufficient to assess long-term outcomes (e.g., patch durability) or late complications (e.g., restenosis or aneurysm formation). The lack of consistent mid- to long-term echocardiographic or catheterization data limits the evaluation of restenosis rates and pressure gradients across repaired segments. No histological analysis of explanted patches was possible as none were removed, precluding insights into tissue remodeling. The absence of a comparison group (e.g., patients treated with synthetic patches, the autologous pericardium, or pulmonary homografts) restricts claims about ProxiCor^®^’s superiority. Additionally, the variability in follow-up durations (Q1–Q3: 363–584 days) due to sequential enrollment may introduce bias as patients with longer follow-up could reveal late complications not yet evident in those with shorter follow-up, potentially skewing the interpretation of early outcomes.

The use of ProxiCor^®^ ECM patches in pediatric cardiac surgery has shown promising results in both aortic arch and pulmonary artery reconstructions, as detailed in our two studies conducted at the Department of Pediatric Cardiac Surgery, Poznan University of Medical Sciences (Poznań, Poland), between 2023 and 2024 [18]. While both studies evaluated the use of ProxiCor^®^ for CHD repairs, the anatomical and hemodynamic differences between aortic arch and pulmonary artery repairs provide a basis for comparative analysis. Notably, the aortic arch repairs often involved complex reconstructions, such as bridging interrupted segments or augmenting hypoplastic arch, requiring multiple patch segments to be sewn together. In contrast, pulmonary artery repairs typically focused on enlarging narrowed segments or reinforcing outflow tracts, which may involve lower mechanical stress. This difference may contribute to the slightly better early outcomes in the pulmonary artery cohort, although the longer follow-up period also allowed for more robust assessment of patch stability. Both studies lacked histological data due to a lack of patch explantations, limiting insights into long-term remodeling differences between high-pressure (aortic) and lower-pressure (pulmonary) environments. ProxiCor^®^ demonstrated superior early performance with no restenosis in either cohort. This may be attributed to its non-crosslinked matrix, which likely enhances cellular repopulation and reduces inflammatory responses, as supported by prior studies on SIS-based scaffolds. However, the distinct hemodynamic contexts—that is, high-pressure systemic flow in aortic repairs versus lower-pressure pulmonary flow—may influence long-term outcomes, with aortic patches potentially facing greater risks of dilation or calcification over time.

The lack of a control group and limited quantitative echocardiographic data (pre/post gradients) prevent direct comparisons and detailed hemodynamic evaluation.

## 5. Conclusions

In this cohort of 25 pediatric patients, ProxiCor^®^ ECM patches appeared to be safe and feasible for pulmonary artery and RVOT reconstruction, with favorable early outcomes. Early postoperative TTE confirmed stable vessel diameters without restenosis or dilation. The 8% mortality rate was unrelated to the patches, reflecting the complexity of the patient cohort. Due to the small cohort size, lack of a control group, and limited follow-up data, these findings are preliminary, and larger, controlled studies are required for confirmation of long-term durability, restenosis rates, and comparative efficacy.

## Figures and Tables

**Table 1:** Demographic and clinical characteristics.

Demography	Parameters	Analyzed Group (*n* = 25)
Sex	Males (*n*) (%)/females (*n*) (%)	17 (68)/8 (32)
Age	1. Newborns (≤28 days of age) (*n*) (%)	4 (16)
	median age (days) (Q1–Q3)	18.0 (15.8–21.3)
	2. Infants (1–12 months of age) (*n*) (%)	15 (60)
	median age (days) (Q1–Q3)	217.0 (171.5–270.5)
	3. Children (>12 months of age) (*n*) (%)	6 (24)
	median age (days) (Q1–Q3)	2442 (888–3091)
Anthropometry	Median height (cm) (Q1–Q3)	68 (62–74)
	Median weight (kg) (Q1–Q3)	6.7 (5.1–8.5)
Primary diagnosis	Tetralogy of Fallot (ToF) (*n*) (%)	6 (24)
	Pulmonary artery stenosis (PS) (*n*) (%)	5 (20)
	Transposition of the great arteries (TGA) (*n*) (%)	3 (12)
	ALCAPA (*n*) (%)	1 (4)
	Univentricular heart (*n*) (%)	10 (40)
Previous surgeries	Systemic-to-pulmonary shunt (*n*)	5
	Norwood procedure (*n*)	3
	Pulmonary artery banding (PAB) (*n*)	2
	Jatene arterial switch operation (ASO) (*n*)	2
	Nikaidoh aortic root translocation (*n*)	1
	RVOT reconstruction (*n*)	1

Abbreviations: ALCAPA—anomalous left coronary artery from the pulmonary artery (Bland–White–Garland syndrome), PAB–pulmonary artery banding (Muller–Dammann procedure), RVOT—right ventricular outflow tract.

**Table 2:** Intra- and postoperative characteristics.

Parameters		Analyzed Group (*n* = 25)
Intraoperative characteristics	Overall surgery time (minutes) (median) (Q1–Q3)	240 (185–270)
	CPB time (minutes) (median) (Q1–Q3)	100 (73–142)
	AoX time (minutes) (median) (Q1–Q3)	68 (59–72)
	Cardioplegia (*n*) (%)	9 (36)
Postoperative characteristics	Mechanical ventilation time (hours) (median) (Q1–Q3)	173 (26–484)
	Prolonged mechanical ventilation * (*n*) (%)	16 (64)
	Continuous renal replacement therapy (CRRT) (*n*) (%)	2 (8)

Abbreviations: AoX—aortic cross-clamp, CPB—cardiopulmonary bypass, CRRT—continuous renal replacement therapy. * >72 h.

**Table 3:** Hospitalization data.

Parameters		Analyzed Group (*n* = 25)
Survival	Overall (*n*) (%)	23 (92)
Mortality	Early * (*n*) (%)	1 (4)
	Late ** (*n*) (%)	1 (4)
	Overall (*n*) (%)	2 (8)
Hospitalization	ICU (hours) (median) (Q1–Q3)	251 (106–825)
	Overall (days) (median) (Q1–Q3)	22 (8.5–38.5)

Abbreviations: ICU—intensive care unit. * ≤30 days after surgery ** >30 days after surgery.

## Data Availability

The data presented in this study are available on request from the corresponding author.

## Abbreviations

The following abbreviations are used in this manuscript:

ABB	Acid–base balance
ALCAPA	Anomalous left coronary artery from the pulmonary artery
AoX	Aortic cross-clamp
ASO	Arterial switch operation
AVB	Atrioventricular block
CHD	Congenital heart defect
CPB	Cardiopulmonary bypass
CRRT	Continuous renal replacement therapy
CVVHDF	Continuous veno-venous hemodiafiltration
ECC	Extracorporeal circulation
ECM	Extracellular matrix
ePTFE	Expanded polytetrafluoroethylene
HLHS	Hypoplastic left heart syndrome
ICU	Intensive care unit
IVC	Inferior vena cava
KROK	Krajowy Rejestr Operacji Kardiochirurgicznych—national cardiac surgery registry
LPA	Left pulmonary artery
MPA	Main pulmonary artery
PA	Pulmonary atresia
PAB	Pulmonary artery banding
PET	polyethylene terephthalate
PS	Pulmonary stenosis
RPA	Right pulmonary artery
RVOT	Right ventricular outflow tract
SIS	Small intestinal submucosa
SVC	Superior vena cava
TGA	Transposition of the great arteries
ToF	Tetralogy of Fallot
TTE	Transthoracic echocardiography
USP	United States Pharmacopeia
VSD	Ventricular septal defect
